# Two Stacked Coins Mimicking an Esophageal Button Battery: A Case Presentation and Review of the Literature

**DOI:** 10.7759/cureus.38795

**Published:** 2023-05-09

**Authors:** Nicholas Rossi, Devin Reddy, John Coggins, Duncan C Watley, Harold S Pine, Shiva Daram

**Affiliations:** 1 Otolaryngology - Head and Neck Surgery, University of Texas Medical Branch, Galveston, USA

**Keywords:** esophagoscopy, radiographic interpretation, foreign body ingestion, coin, button battery

## Abstract

As button battery (BB) ingestion has become a popular topic with growing public awareness in recent years, pediatric otolaryngologists maintain a high index of suspicion for this diagnosis. Several recent reports have revealed the possibility for benign objects to masquerade as BBs, such as two coins stacked together or a coin with different metals in concentric rings. A 4-year-old female presented to the ED after unwitnessed ingestion of a foreign body. The child was reportedly seen playing with her sister’s coin collection prior to the acute onset of drooling and dysphagia. She was vitally stable and without any shortness of breath, stridor, or wheezing. Plain film X-ray revealed a round, metallic object with a double density on the frontal view and beveled step-off on the lateral view at the level of the thoracic inlet. Due to high radiographic concern for BB ingestion, the patient was taken emergently to the operating room for a rigid esophagoscopy. A metallic object was seen at the thoracic inlet and removed with Magill forceps. The object was found to be two coins stuck together, with a smaller coin in the center of a larger coin mimicking the shape of a BB. The patient was discharged the next day without complication. This case highlights stacked coins as a radiologic masquerade for BBs as well as the emphasis on prompt esophagoscopy for both identification and removal. Radiographic densities alone cannot be relied upon to distinguish BBs from more innocuous objects, and esophagoscopy remains the mainstay of management for pediatric esophageal foreign bodies.

## Introduction

Foreign body ingestion (FBI) is common in the pediatric emergency setting, with coins responsible for 60-85% of cases [1]. With at least 40% of all pediatric FBIs being unwitnessed, clinicians are heavily reliant on a good history and imaging studies to identify the foreign body [2]. Button batteries (BBs) are disc-shaped batteries used primarily in watches and car keys. They appear similar to coins on radiographic imaging. BBs make up to 2% of pediatric FBIs, and there has been a significant increase in morbidity and mortality due to their ingestion in the past two decades [1,3]. Children younger than five are at the greatest risk of severe injury due to BB ingestion, making any suspected ingestion a prioritized emergency.

Upon ingestion of a BB, the battery can become impacted in the esophagus causing pressure necrosis, electrical burns, and caustic burns via the production of hydroxide free radicals, resulting in liquefactive necrosis. Within 15 minutes of ingestion, there is visible damage to the mucosa and serious injury after as little as two hours. Further complications may present weeks or months after BB removal, including esophageal perforation, tracheoesophageal fistula, vocal cord paresis and paralysis, tracheal stenosis, tracheomalacia, mediastinitis, vascular injury, and death [3]. At least 90% of serious outcomes caused by BB ingestion were due to the larger (20mm+), newer, and more popular lithium-ion cells, which are more powerful than their older alkaline predecessors and more likely to become impacted [3,4].

Although coins are the most common cause of pediatric FBIs, BBs are potentially catastrophic if left lodged in the esophagus; therefore, differentiating coins from BBs on imaging is essential. Plain radiographs are typically sufficient to distinguish coins from BBs based on the raised central portion and varying densities of a BB. This appearance is easily replicated by two coins stacked together, as presented in the following case.

## Case presentation

**Figure 1:**
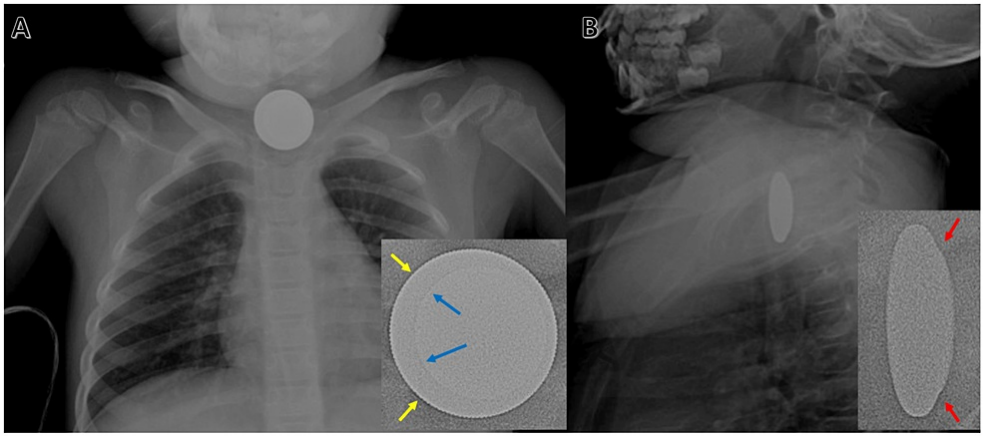
Radiographic images of two stacked coins Image A shows an anterior-posterior radiograph (kilovoltage peak (KVP) 75, milliampere-seconds (mAs) 1) of the chest demonstrating a round, metallic object in the distal cervical esophagus at the level of the thoracic inlet. The magnified inset image uses the Edge Enhance feature of the Picture Archive and Communication System. Blue arrows indicate the edge of the penny on the quarter which resembles closely the rim of a BB. Yellow arrows indicate the reeded edge of the quarter. Image B shows a lateral radiograph (KVP 80, mAs 3) of the neck demonstrating an object with a beveled appearance resembling a BB. Red arrows in the magnified inset image indicate the bevel between the penny and the quarter

A 4-year-old girl presented to an outside hospital ED with her mother after unwitnessed ingestion of a foreign body. She was vitally stable with a heart rate of 132 beats per minute, blood pressure of 118/79 mmHg, and respiratory rate of 28 breaths per minute. She endorsed drooling and dysphagia but was without any shortness of breath, stridor, or wheezing. A plain film X-ray revealed a round, metallic object in the distal cervical esophagus at the level of the thoracic inlet (Figure 1). On frontal view, the object appeared to have a faint ring near the outer edge indicative of a “halo sign” or “double-ring sign.” On the lateral view, the object had a beveled appearance similar to that of a BB. At this point, the patient was at an outside ED without access to specialty care, and transport to our academic facility would take a minimum of 30 minutes via helicopter. With the stability of the airway of utmost concern, the decision was made to proceed with the transport of the patient to the main hospital for urgent treatment given the high radiographic suspicion of BB ingestion.

Upon arrival, the patient was again clinically and vitally stable. Obtainment of further history revealed that the child was reportedly seen playing with her sister’s coin collection prior to the acute onset of symptoms earlier that evening. She was then transported emergently to the operating room for a rigid esophagoscopy. In the operating room, she was laid supine in the usual fashion, and a rigid esophagoscope was gently inserted transorally. Two stacked metallic objects were seen lodged against the esophageal mucosa just distal to the level of the upper esophageal sphincter. The objects were carefully removed with Magill forceps. The objects were discovered to be a quarter and a penny stuck together, with the penny in the center of the quarter mimicking the shape of a BB. Esophagoscopy was repeated to evaluate the mucosal integrity of the esophagus, which was found to be without mucosal laceration or perforation. The patient was observed overnight and monitored for oral intake and signs of mediastinitis. She was discharged the next day without complication.

## Discussion

For patients with an FBI, the standard of practice is to order both anterior-posterior and lateral radiographs. The step-off and differences in height between the positive and negative nodes of the cell give BBs their two most characteristic features: on AP view, a “double-halo sign” around the outer region of the disc, and a raised center portion on lateral view [1,3]. Radiographs may have a strong negative predictive value of BB ingestion, especially when coupled with a negative medical history [5]. Due to the variety of BBs available on the market, not all BBs have easily distinguishable double-halos on radiographs due to differences in sizing dimensions and step-off effect [1,6]. Various methods have been developed to aid quick and accurate diagnosis, although with limited success. These include artificial intelligence, the use of reverse contrast on the radiograph, improved radiograph resolution, contrast and dynamics, and digital image analysis [1,7,8].

The distinction between BBs and coins on radiographs can be further blurred in the cases of stacked coin ingestion (as is the case with our patient) or dual-metallic coin ingestion. On radiographs, both also will show the double-halo sign, and it has been suggested that radiographic density nor size no longer provides a reliable measure in distinguishing between the coin(s) and BBs [2,6]. In a study done by Safavi et al. in 2016, the researchers reported that the Japanese 1 yen and South American pesos were most confused for BBs [1]. The peso (e.g., the Mexican five peso coin) may be constructed of two different metals, forming inner and outer rings of incongruent densities on radiographs. The Japanese 1 yen is constructed from aluminum, significantly more radiolucent on radiographs than other coins.

If BB ingestion is even remotely suspected, the patient is emergently taken to the operating room for endoscopic evaluation and foreign body removal. Postoperative management is contingent on intraoperative esophageal appearance and institutional policy. Management can include placement of a nasogastric tube (intraoperatively), repeat MRIs to monitor possible complications, a 2-4-week course of proton pump inhibitors, antibiotics, and an esophagram prior to resuming a normal diet [2,9]. Since significant injury can occur very quickly upon BB ingestion, the use of level I trauma activation has been found to lower the time to evaluate and remove BBs [3].

Cases of esophageal BB impaction are rare, making gaining substantial experience in identifying and managing the injury difficult. Incorrectly identifying a BB as a coin results in delays in diagnosis, treatment, and catastrophic esophageal injury. Quickly recognizing BBs and rapid removal are critical in minimizing possible complications.

## Conclusions

Children are prone to ingesting coins and batteries which may become lodged in the esophagus or airway. Batteries are a medical emergency due to their ability to burn and erode through the esophagus. Plain radiographs are typically capable of distinguishing coins from BBs due to the step-off and hyperdense outer ring; however, this case demonstrates that these findings may be mimicked by two coins stacked within the esophagus. If a BB is suspected, an emergent esophagoscopy should be conducted to remove the battery.
